# Compounds from Natural Sources as Protein Kinase Inhibitors

**DOI:** 10.3390/biom10111546

**Published:** 2020-11-12

**Authors:** Andrea Baier, Ryszard Szyszka

**Affiliations:** 1 Department of Animal Physiology and Toxicology, Institute of Biological Sciences, The John Paul II Catholic University of Lublin, 20-950 Lublin, Poland; 2 Department of Molecular Biology, Institute of Biological Sciences, The John Paul II Catholic University of Lublin, 20-950 Lublin, Poland; szyszkar@kul.pl

**Keywords:** protein kinases, inhibitors, natural compounds

## Abstract

The advantage of natural compounds is their lower number of side-effects when compared to most synthetic substances. Therefore, over the past several decades, the interest in naturally occurring compounds is increasing in the search for new potent drugs. Natural compounds are playing an important role as a starting point when developing new selective compounds against different diseases. Protein kinases play a huge role in several diseases, like cancers, neurodegenerative diseases, microbial infections, or inflammations. In this review, we give a comprehensive view of natural compounds, which are/were the parent compounds in the development of more potent substances using computational analysis and SAR studies.

## 1. Protein Kinases as Therapeutic Targets

**Figure 1:**
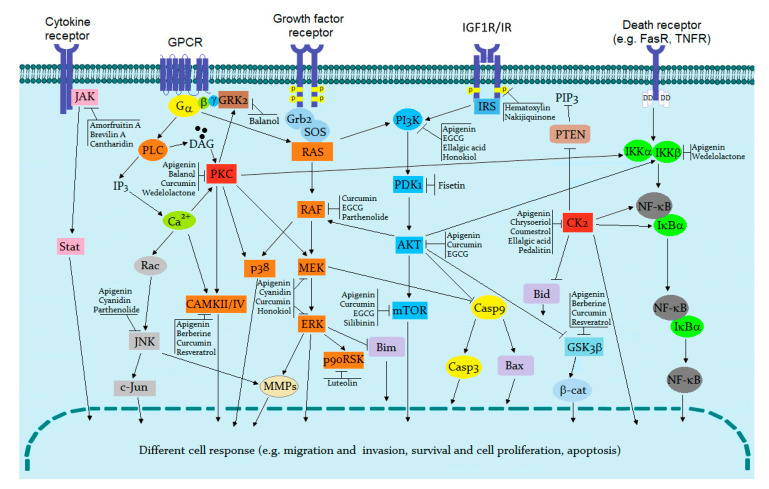
Schematic presentation of intracellular transduction pathways, including protein kinases and examples of their inhibition by natural compounds.

Protein phosphorylation is one of the most frequent and significant post-translational modifications [1,2]. Protein kinases, which are nearly all members of the eukaryotic protein kinase (ePK) superfamily, represent a large and diverse family of enzymes. They catalyze covalent modification of proteins by attaching phosphate groups (from ATP or GTP) mainly to serine, threonine, and/or tyrosine residues. Phosphorylation has many effects on proteins. The added charge and bulk of the phosphate group modifies the protein from hydrophobic apolar to hydrophobic polar. This may result in changes in protein conformation and/or alter the enzyme activity and, therefore, influence the interaction between proteins, localization, or the degradation of protein substrates. Protein kinases catalyzing the process of phosphorylation are base elements of cell signaling transduction cascades, such as the control of cell growth and proliferation, response to extracellular stimuli, DNA damage response, control of metabolism, and death. Reversible protein phosphorylation is the main strategy for the control of eukaryotic cell activities. Figure 1 presents an overview of signaling pathways, the protein kinases involved, and the potential targets of natural substances.

The first complete study of human kinases identified and classified 518 protein kinases by grouping them into evolutionary related families based on statistical sequence analysis and biological function [3,4,5]. In addition, this analysis showed that 478 of them possessed catalytic domains with related primary sequences. Thirty-two atypical kinases (e.g., PI3K, RIO kinase) with dissenting primary sequences have been identified [3]. In a later study, two novel kinases were added that contained an interesting combination of kinase and acyl-CoA dehydrogenase domains [6]. Subsequent analysis of the human kinome led to the identification of 23 new kinases and removed three for a total of 538 protein kinases [6,7,8]. A recent reassessment of the human kinase superfamily showed 555 members grouped into main classes of 497 eukaryotic protein kinases (ePKs) and 58 atypical protein kinases (aPKs), due to lack of sequence similarity [9]. This shows that gene encoding for protein kinases make up almost 2% of the human genome and control essential biological processes.

The 555 human protein kinases (KinBase: www.kinase.com/web/current/kinbase/) phosphorylate either serine/threonine (Ser-/Thr-specific protein kinases; S/T PKs), tyrosine (Tyr-specific protein kinases; T PKs), or both Ser/Thr as well as Tyr (dual-specificity protein kinases) [1,2,3,4]. A total of 497 protein kinases have a common catalytic site, which contains a glycine-rich N-terminal ATP-binding pocket and a conserved central aspartic acid residue required for catalytic activity [4,5]. The ePK class is further subdivided on the basis of primary sequences into nine major groups, and each group is then split into families [3,4]. In Table 1, a number of eukaryotic protein kinases are listed, and their biological functions are given.

The AGC kinase group of protein kinases contains 64 members and can be classified into 14 subfamilies, including PKA, PKG, and PKC. The family comprises some intensively examined protein kinases (such as Akt/PKB, S6K, RSK, MSK, PDK1, and GRK) [63]. A unique feature of AGC kinases is the presence of a C-terminal segment containing a hydrophobic motif within the catalytic domain; whereas, selectivity and specificity in the regulation of AGC kinases are predominantly derived from the regions located N- and C-terminally to the catalytic site.

The CAMK group is named after calcium and calmodulin-regulated kinases and contains 82 members. This subfamily of S/T PKs translates and coordinates an increase of intracellular Ca^2+^ concentration into cellular responses via phosphorylation. The activity of this kinase is regulated primarily by the Ca^2+^ receptor protein calmodulin (CaM). This family includes CAMKI-V, and CAMK kinase 1 and 2, and is dedicated to single substrates: phosphorylase kinase (PhK), myosin light chain kinase (MLCK), and eukaryotic elongation factor-2 kinase (eEF2K).

The CK1 group (known as casein kinase 1) is a small group of kinases (12 members) that are very similar to each other in sequence, but very distinct from other kinase groups. This group represents ePKs involved in many diverse and important cellular functions, such as the regulation of membrane transport, cell division, DNA repair, and nuclear localization.

The CMGC group includes key kinases: the MAPK growth- and stress-response kinases, the cell cycle CDK (cyclin-dependent kinases), and kinases involved in splicing and metabolic control. There are nine highly conserved CMGC families: CDK, CDK-like, CK2, CLK, DYRK, GSK, RCK, SRPK, and MAPK. These are the final kinases in the MAPK cascade that link extracellular signals, including growth factors and stress signals, to transcriptional and other responses, e.g., the ERK superfamily, p38, or JNK. The CMGC group contains 65 members.

The STE group consists of three main families (49 members in total), including homologs of the yeast sterile kinase sterile 7 (Ste7), sterile 11 (Ste11), and sterile 20 (Ste20), which successively activate each other and MAPK members. The Ste7 family phosphorylates MAPK directly, and is also known as MEK; members of the Ste11 family phosphorylate Ste 7 kinases, while many Ste20 members act on Ste11 kinases. Distinct sets of Ste7 and Ste11 kinases are associated with specific classes of “executive” MAPK (ERK, JNK, p38, and others). Some cross-talk is also seen. Ste7 activity is also found in the tyrosine kinase-like family (TKL) in kinases as RAF and MLK, as well as in other kinase families.

The Other kinase group consists of 82 kinases with an ePK domain that does not fit into any of the other major groups. Kinases belonging to the Aurora family (Aur) are mitotic kinases, involved in centrosome and cilia biology; CAM kinase kinase (CAMKK) activates CAMK1 upon calmodulin-binding, which is known to be an upstream kinase for the AMPK kinases.

The RGC kinases are represented by a small ePK group of five receptor guanylate cyclases similar in sequence to tyrosine kinases found in metazoa but absent in fungi, plants, and protists. The RGC group, therefore, represents a late innovation in the protein kinase superfamily. The photoreceptor membrane guanylate cyclase domain possesses Mg^2+^-dependent serine auto-phosphorylating kinase activity [64]. This kinase activity is unaffected by Ca^2+^, cyclic nucleotides and phorbols, but is inhibited by high concentrations of staurosporine. These properties differ from other serine/threonine kinases.

The TK (tyrosine kinase) group is divided into two large classes: receptor and non-receptor TKs. Among the 95 identified human tyrosine kinase genes, 58 encode for receptor tyrosine kinases (RTKs), which are divided into 20 subfamilies [65,66,67,68]. The RTKs include the insulin receptor families (IGFR) [69] and other growth factor receptors, such as epidermal growth factor (EGFR, Her2/neu, Her3, and Her4) [21], platelet-derived growth factor (PDGFR) [70], fibroblast growth factor (FGFR) [71], vascular endothelial growth factor (VEGF) [72], and nerve growth factor (NGF) [73]. RTKs are single-pass, type I receptors that are anchored in the plasma membrane. Generally, RTKs are activated through ligand-induced oligomerization, typically dimerization, which connects the cytoplasmic tyrosine kinase domains. For most RTKs, this juxtaposition facilitates the trans-autophosphorylation of tyrosine residues in the kinase activation loop or juxtamembrane region, inducing conformational changes that stabilize the active state of the kinase. These and other phosphotyrosine residues serve as recruitment sites for a host of downstream signaling proteins—enzymes and adapter/scaffolding proteins—typically through SH2 (Src homology-2) or phosphotyrosine-binding domains, which recognize phosphotyrosine residues in specific sequence contexts. Specific proteins containing these domains include Src and PLCγ. Phosphorylation and activation of them on receptor binding leads to the initiation of signal transduction pathways.

Unlike the RTKs, the second subgroup of tyrosine kinases, the non-receptor tyrosine kinases (nRTKs), are localized in the cytoplasm or, in some cases, are anchored to the cell membrane through amino-terminal modification. Thirty-two nRTKs have been identified in human cells and grouped into 10 subfamilies. These include ABL, FES, JAK (e.g., JAK1-3, TYK2), ACK, SYK, TEC, FAK (e.g., FAK/PTK2), SRC (e.g., Src, Lck, Fyn), and the CSK family of kinases [74,75]. 

The TKL (tyrosine kinase-like) kinases are a diversified group of Ser/Thr protein kinases with sequence similarity to TKs but lacking specific motifs for tyrosine kinases. Families in this group are relatively weakly related to each other. TKLs are the largest group of kinases in plants, where they often constitute around 80% of the plant kinome. There are eight major TKL families in animals. Mixed-lineage kinases (MLK) are named due to their similarities of sequences to both Ser/Thr and Tyr protein kinases. They frequently act as a first element in MAPK cascades. For example, MLKs are known to be responsible for activating apoptotic pathways implicated in the pathogenesis of Parkinson’s disease [76]. The TGFβ type-I receptor activin receptor-like kinase 1 (ALK1) and its co-receptor endoglin play fundamental roles in angiogenesis and vascular development [77].

The Atypical kinase (aPK) group (58 members in total) represents protein kinases (or putative PKs) that do not share clear sequence similarities with conventional ePKs, but they (or their orthologs) possess protein kinase activity [3,9]. Although ePKs and aPKs are highly divergent on the primary sequence level, at the tertiary level, the structures of their catalytic domains are highly conserved in both the N-lobe and the C-lobe. The conserved glycines in the G-rich loop (GxGxxG) are important for the catalytic activity of eukaryotic kinases, which is missing in the aPKs. Another major difference is observed at the HRD motif, where histidine and arginine are mirrored around the aspartate, creating the atypical DRH motif [9].

One of the families of aPKs is represented by phosphatidylinositol 3-kinase-related kinases (PIKK). PI3K catalyzes phosphorylation of phosphoinositide (PI) at position three and plays a key role in a variety of cellular functions. It is stimulated by both GPCR and RTKs. PI3Kγ is specifically activated through the direct interaction with the βγ subunit of G-protein. PI3Kβ is synergistically activated by the βγ subunit and phosphotyrosine-containing peptide. Both PI3K α and δ are activated by the TK pathway but not by G-protein-mediated pathway. In particular, the PI3Ks, which phosphorylate PI together with another member of the PI3K-related kinase family—mTOR, have been implicated in cancer and immunological disorders [78,79,80]. 

The protein kinase family offers a challenge but also an enormous opportunity for drug discovery. Disease arises when signal transduction in a cell breaks down, thereby removing the tight control that typically exists over cellular functions. Devastating diseases, such as cancer, neurological disorders, inflammation, autoimmune diseases, psoriasis, allergic reactions, and hormone-related diseases, can result from abnormal signal transduction.

Protein kinases, as key regulators of most cellular pathways, are frequently associated with diseases, either as triggers or as therapeutic intervention points. Several small molecule drugs targeting kinases (mostly receptor tyrosine kinases) are already on the market, mostly as anti-cancer therapeutics, and hundreds of kinase inhibitors are in various stages of development [78,79,80,81,82,83,84]. 

## 2. Classes of Protein Kinase Inhibitors

Protein kinase inhibitors are categorized according to their mechanism of action. In the beginning, small molecule protein kinase inhibitors were divided into three classes, namely type I, II, and III kinase inhibitors [85]. Type I kinase inhibitors are molecules which bind to the ATP pocket in the active conformation of a kinase and alter the structural conformation otherwise favorable to phosphotransfer [86,87], whereas the type II inhibitors bind to an inactive conformation of a kinase and interact with the catalytic site of the unphosphorylated inactive conformation of kinases [88]. 

Inhibitors of type I are typically characterized by a heterocyclic ring system that occupies the purine binding site through mimicking the purine ring of the adenine moiety of ATP [89]. They directly interact with the conformational phosphorylated active catalytic site of the kinases.

Type II kinase inhibitors bind inside the lipophilic pocket, which occurs after changing the conformation of the “DFG” N-terminal loop [88,90]. This kind of interaction is reversible and leads to single or multiple hydrogen bonds within the “hinge region” [86,88].

Type III inhibitors were initially classified as non-ATP competitive or allosteric inhibitors [85,91,92]. Soon after, the allosteric inhibitors were divided into classes III and IV [93]. While type III inhibitors are described as acting within the cleft between the small and large lobes close to the ATP binding pocket, the type IV molecules bind outside of the cleft in the phosphor-acceptor region. Inhibitors of class III are steady-state non-competitive or uncompetitive inhibitors with respect to the phosphate donor. This means higher concentrations of ATP cannot prevent this interaction between the molecule and the kinase.

Type IV molecules reversibly bind to the substrate-binding site outside the ATP pocket. Therefore, they do not compete with ATP and are substrate-directed inhibitors [94].

Later on, compounds that reacted with the enzyme by covalent interactions were termed type V [91,95]. The covalent bond between the inhibitors and the kinase active site is irreversible [96,97], and the target for these inhibitors is the exposed cysteine side chain in the ATP site [98,99].

As potential drug molecules within these classes differ in their selectivity and safety, class I inhibitors are less selective due to the highly conserved ATP pocket through the kinome to which they bind. Type II compounds show a higher selectivity profile because they bind to the inactive kinase conformation, which is less conserved within the kinome [88,89]. Limited selectivity in the case of kinase inhibitors might be avoided by developing allosteric inhibitors. By using those molecules, other problems, like off-target side effects and drug resistance, can be also solved.

More detailed information about protein kinase inhibitors, their classification, and mechanisms of action are given in [81,100].

## 3. Natural Compounds as Kinase Inhibitors

During the last decades, a lot of research groups worldwide have developed different strategies to fight against human diseases like cancers, neurological, or auto-immunological disorders. Besides employing chemically synthesized molecules as protein kinase inhibitors, in recent years, a huge amount of natural compounds were described as the starting point for new substances acting as protein kinase inhibitors, and, therefore, potential drug candidates.

Table 2 summarizes different natural compounds regarding their target kinase and biological activities.

### 3.1. Polyphenol Analogues

**Figure 2:**
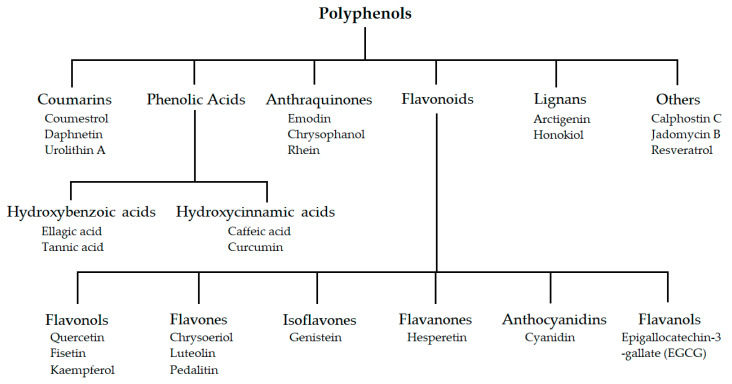
Classification of polyphenolic compounds.

The class of polyphenols can be divided into several major groups like phenolic acids, flavonoids, anthraquinones, coumarins, and lignans. Polyphenols are a structural class characterized by the presence of several phenol units. The classifications and few naturally occurring members are presented in Figure 2. Polyphenols exhibit various therapeutic effects in numerous diseases, like cancers, inflammations, cardiovascular, and neurological diseases.

#### 3.1.1. Flavonoid Analogues

**Figure 3:**
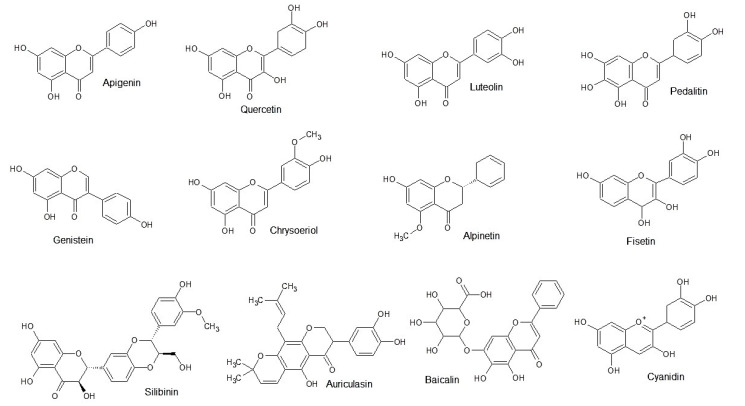
Chemical structures of selected flavonoids.

Flavonoids are a class of natural substances that can be divided into seven different groups: flavanones, flavones, isoflavones, flavanols, flavonols, flavanolones, and anthocyanins. Flavonoids occur in several fruits and vegetables and belong to important phytochemicals used in the prevention and therapy of diverse diseases. Examples of some flavonoids are shown in Figure 3. As will be described, flavonoids possess a broad spectrum activity towards protein kinases, showing less specificity towards a specific class of kinases.

Quercetin naturally occurs in different fruits and vegetables, such as apples, grapes, berries, and onions. Besides others, it is one of the earliest described flavonoids possessing inhibitory activity towards protein kinases [141]. It has been shown that quercetin acts on multiple kinase targets, which are mostly involved in cell proliferation in cancer cells. The PI3K-Akt/PKB pathway is inhibited through the binding of quercetin to PI3Kγ (IC_50_ = 3.8 µM) without targeting Akt/PKB [142,143]. Another kinase participating in the same pathway is CK2. Quercetin is a well known ATP-competitive CK2 inhibitor that is often used as a standard in kinetic assays. CK2 is inhibited with IC_50_ values between 0.2 and 1.8 µM, depending on the examined isoform and phosphorylated protein substrate [144,145,146,147]. Other kinases, including MEK-1, GSK-3β, Hck, and IKKα/β, have also been found to be inhibited at low micromolar levels. In the case of Hck and GSK-3β, quercetin binds to the ATP-binding pocket with an IC_50_ value of 2 µM [148,149]. On the other hand, MEK-1 is inhibited through a different mechanism, which is the binding of quercetin to the activation loop situated adjacent to the ATP-binding pocket [142,150]. Within these kinases, quercetin exhibits the weakest effect towards IKKα and IKKβ with IC_50_ values of 11 and 4 µM [151]. Kinetic analysis has demonstrated that the inhibition mode might be via binding to the ATP-binding pocket and the protein substrate binding site. In other studies, quercetin was shown to have less inhibitory potential towards PKC isoforms and MAPK with an inhibition between 9 and 24% at an inhibitor concentration of 50 µM [152]. The same study described quercetin as a substantial inhibitor for tyrosine kinases, such as Syk, Src, Fyn, and Lyn [152]. Within this study, genistein, an isoflavone produced by *Pseudomonas* sp., was tested against a panel of protein kinases representing tyrosine as well as serine/threonine kinases. Genistein exerted a very weak effect against the tested kinases [152]. A further study described genistein as a specific tyrosine kinase inhibitor, which was confirmed by several other research groups [153,154,155]. Genistein exerted inhibitory efficacy against the EGFR kinases (IC_50_ = 22 µM), Src (IC_50_ = 26 µM), and MEK4 (IC_50_ = 0.4 µM) in an ATP-competitive manner [154,155].

Other flavonoids were also described as very potent inhibitors of Ser/Thr protein kinases, like apigenin, chrysoeriol, luteolin, pedalitin, fisetin, and alpinetin. Compared to quercetin, apigenin and luteolin had similar or slightly better effects on GSK-3β [149]. From such results, it can be predicted that flavonoids with larger side chains have lower activity on GSK-3β. Agullo et al. [156] compared the activity of several flavones and flavonols on PI3K, EGFR kinase, and PKC. Both serine/threonine kinases, PI3K and PKC, were inhibited by a similar profile. The most hydroxylated flavonoids (myricetin, quercetin, and luteolin) were the most potent inhibitors, whereas compounds with only two hydroxyl groups (galangin and chrysin) possessed almost no effect on the kinase activities. These findings did not hold true for EGFR tyrosine kinase. The observed structure–activity relationship was opposite to that of PI3K and PKC [156]. Flavonoid compounds with less hydroxyl groups (apigenin and kaempferol) showed higher inhibitory potential towards EGFR kinase than quercetin and luteolin.

The first results using flavonoids as protein kinase CK2 inhibitors were described by Li et al. and Lolli et al. [144,145]. As we have demonstrated in our former publications, flavonoids isolated from plant materials are potential inhibitors of protein kinase CK2. We tested more than 20 compounds (e.g., apigenin, pedalitin, and chrysoeriol) for their effect on different CK2 isoforms and showed that CK2α’ was the most sensitive towards the examined compounds [146,147]. Chrysoeriol was found to be the most promising flavonoid, with IC_50_ values in the low nanomolar range. Most other tested flavonoids possessed an inhibitory effect, with IC_50_ values between 0.2 and 10 µM for free catalytic CK2 subunits and between 1 and 20 µM for the CK2 holoenzymes.

Quercetagetin, the major flavonoid in marigold, was characterized as a potent and selective inhibitor of PIM1 kinase [131]. It was shown that the IC_50_ value for PIM1 was 0.34 µM, while the respective values for other tested serine/threonine kinases (c-Jun, PKA, Aurora-A, c-Raf, and PKC) were 9- to 70-fold higher. Besides quercetagetin, other flavonoids, like gossypin, myricetin, fisetin, and apigenin, decreased the PIM1 activity with IC_50_ values below 1 µM. Quercetin, kaempferol, luteolin, and morin still showed very good effects on the kinase activity with IC_50_ values between 1 and 3 µM.

Cyclin-dependent kinases (CDKs) are involved in the regulation of cell cycles and transcription. Several flavonoids have been found to inhibit CDK9. The most potent inhibitor with an IC_50_ value in nanomolar concentrations (190 nM) was found to be wogonin, which also inhibited CDK2 and 7, but at much higher concentrations of 1.46 and 12.3 µM, respectively [157]. Other flavonoids showed weaker effects on CDK1, 5, and 6, as summarized by Jain et al. [158]. Genistein was ineffective when tested against CDK2 and 4, whereas fisetin was detected to reduce the CDK1, 5, and 6 activities with IC_50_ values below 1 µM. Kaempferol and quercetin, being good inhibitors in cases of other serine/threonine kinases, showed weak effect towards CDK 1, 5, and 6, as well as CDK2 and 4, respectively, with IC_50_ values between 20 and 60 µM. Luteolin, apigenin and chrysin were identified as good inhibitors, with values below 10 µM.

#### 3.1.2. Phenolic Acids

**Figure 4:**
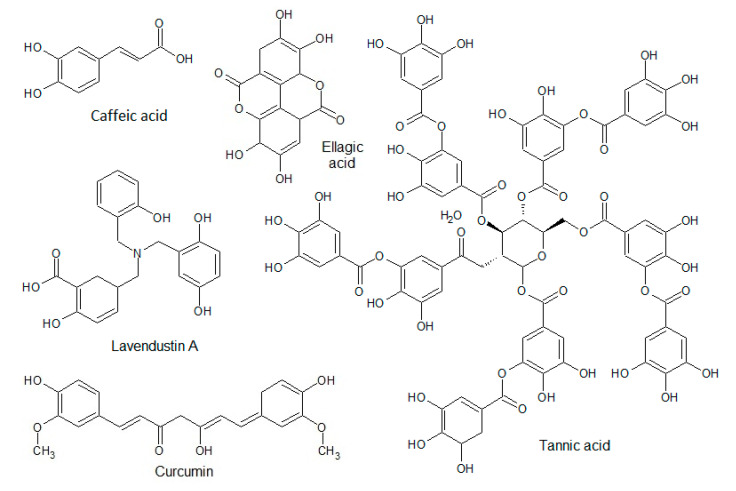
Structures of phenolic acids.

Within the class of polyphenolic compounds, 30% belong to phenolic acids. They are divided into two major groups: hydroxybenzoic and hydroxycinnamic acids. Hydroxybenzoic acids are simple, aromatic acids possessing strong antioxidative and anticancer activities. The most important representatives are tannic, gallic, and ellagic acid. Figure 4 shows the structures of some members of that class. 

Tannic acid is an ingredient of tara, nutgall tree, Aleppo oak, and Sicilian sumac. It was shown that a concentration of 16 μM decreased the activities of conventional PKC isoforms α, βI, and βII in mouse epidermal cell line [159]. The possible mechanism of action was examined by Kashiwada et al., and indicated a competitive action towards phorbol ester, with IC_50_ values in the low micromolar range. This demonstrated that tannic acid might bind to the regulatory domain of PKC [160,161].

Ellagic acid is a dimer of gallic acid and can be found in several fruits and vegetables. It was identified as a very potent CK2 inhibitor using a virtual screening application with a Ki value of 20 nM [162]. In a further study, to analyze the selectivity towards CK2, the same authors tested ellagic acid on a total of 70 protein kinases [163]. At a concentration of 10 µM, few kinases were better inhibited than CK2: PRAK, SRPK1, DYRK3, and DYRK2. The residual activity of 13 kinases was 30% or below. Numerous protein kinases did not show decreased activity, like MAPK, ERK1, JNK3, and PRK2. Some kinases were even activated, like SmMLCK, CAMKKa, CHK2, and EFK2 [163]. 

Lavendustin A is isolated from *Streptomyces griseolavendus*. It was found to be a very potent and cell-permeable ATP-competitive EGFR inhibitor (IC_50_ = 11 nM) [164,165]. It was also found that lavendustin A potently inhibited Src kinase (IC_50_ = 500 nM), whereas it completely failed against serine/threonine kinases PKA, PKC, and PI3K [166].

Hydroxycinnamic acids are a diverse group of phenolic substances with caffeic and ferulic acid as some of its members. Curcumin is a dimeric derivative of ferulic acid and might also be classified herein.

Caffeic acid can be found in fruits, vegetables, wine, olive oil, and coffee. The potential effect of this simple hydroxycinnamic acid was examined on phosphorylase kinase (PhK), PKA, and PKC [167]. The resulting IC_50_ values revealed a very weak inhibitory effect with values of 80, 95, and 180 µM for PhK, PKC, and PKA, respectively. Despite the low effect on these protein kinases, caffeic acid was further tested as an inhibitor of Fyn kinase, ERK2, JNK1, and p38 [168]. Only Fyn kinase was inhibited by caffeic acid with an IC_50_ of 10 µM. 

Curcumin, the main component of turmeric, is one of the best-characterized anti-cancer agents. The therapeutic effect of curcumin results from its structural features and its unique physico-chemical properties. It was shown that curcumin exerts its effect on PKC in different ways depending on the presence of membranes. Detailed kinetic analysis underlined the fact that this compound competed for the Ca^2+^-binding site, resulting either in inhibition or activation of the activity depending on lower or higher Ca^2+^ concentrations, respectively. In the presence of membranes, the Ca^2+^ was bound; therefore, the kinase activity was decreased. The opposite was observed when Ca^2+^-independent isoforms were examined. Curcumin slightly stimulated the activity [169]. Another work described the influence of curcumin towards PKC through binding to the αC1 domain [170]. This interaction inhibited the phorbol ester-induced membrane translocation of PKCα or PKCε mutants containing the αC1 domain. A further study characterized curcumin as a highly potent inhibitor of DYRK2 with off-target effects on related DYRK isoforms [171]. It was also reported that DYRK2 was inhibited with an IC_50_ of 5 nM in vitro. To evaluate the specificity of curcumin, an additional 140 protein kinases were examined, including closely related enzymes from the CMGC kinase family. As shown, curcumin targeted DYRK2 specifically. Other kinases, like DYRK1A, DYRK3, PIM, MLK, and PhK, were also inhibited but evidently to a lesser extent compared with DYRK2. DYRK isoforms 1A and 3 were inhibited at low nanomolar concentrations, whereas PIM, MLK, and PhK showed reduced activity with IC_50_ values still below 1 μM. An in vitro effect on purified IKKβ (IC_50_ > 10 μM) and GSK3β (IC_50_ > 3 μM) was detected only at higher (micromolar) concentrations.

#### 3.1.3. Anthraquinones

**Figure 5:**
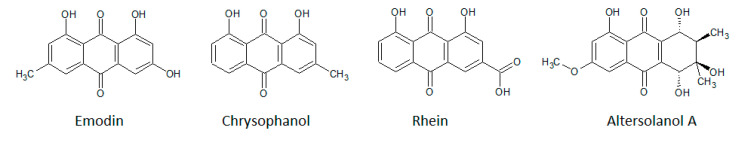
Structures of some anthraquinone derivatives.

Anthraquinones can be found in different organisms: plants, animals, and microorganisms. Figure 5 represents the structures of some anthraquinones.

Emodin, first isolated from the Chinese medicinal plant *Polygonum cuspidatum*, was described as an ATP -competitive inhibitor of tyrosine kinase p56(lck) with an IC_50_ of 18.5 µM [172]. Experiments performed in human breast cancer MDA-MB 453 cells demonstrated that emodin inhibits Her-2/neu kinase with an IC_50_ of 21 µM [173]. A few years later, emodin isolated from the rhizomes of *Rheum palmatum* was also shown to be a potent inhibitor of CK2 with an IC_50_ of 2 µM, whereas it was ineffective towards other serine/threonine kinases, like PKA, PKC, and cdc2 [174]. Later, this anthraquinone was identified as a potent inhibitor of FIKK, a serine/threonine kinase from *Plasmodium vivax*, a parasite responsible for most malaria cases in humans [175]. Emodin was more potent when compared to the p56(lck) and similar to CK2 with an IC_50_ value of 1.9 µM.

Chrysophanol is found in *Rheum* spp. and is used in Chinese medicine. It was shown that chrysophanol inhibits the EGF-induced EGFR phosphorylation and suppresses the activation of downstream Akt and ERK1/2 molecules in SNU-C5 human colon cancer cells [176]. Further research has demonstrated that chrysophanol is involved in several signaling pathways, like PI3K/Akt, MAPK/PI3K, JAK2/STAT3, JNK/p38, and Akt/MAPK [177].

Rhein is isolated from rhubarb and has been used in traditional Chinese medicine to treat constipation, gastrointestinal hemorrhage, and ulcers. Rhein exerts anti- and pro-inflammatory activities by targeting IKKβ inhibition. The IC_50_ value towards IKKβ is 11.79 µM [178]. Furthermore, several pathways or proteins regulating cell survival are possible targets of rhein. Those proteins are serine/threonine kinases, like Akt kinase, CDK, and MAPK (MEK, ERK) [179]. 

Altersolanol A is a metabolite isolated from the fungus *Stemphylium globuliferum*. It has been shown to be a non-specific protein kinase inhibitor, being able to decrease the activity of several kinases, but only in a low micromolar range [180]. Aurora kinases were inhibited with IC_50_ values between 2 and 4 µM, whereas VEGFR kinases were inhibited with 10-times lower values. CDK4 was the most sensitive kinase with an IC_50_ below 2 µM. Altersolanol A did not show any effect towards CK2α, COT, or ERBB2 at concentrations of 30 µM. 

#### 3.1.4. Coumarins

**Figure 6:**
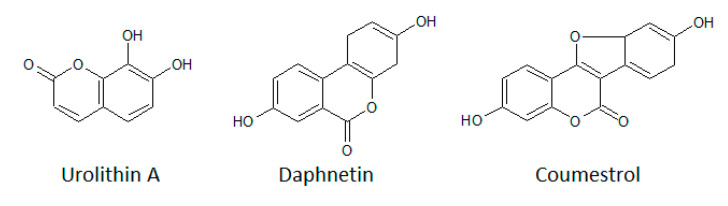
Structures of coumarin derivatives.

Coumarins are secondary metabolites of higher plants and some microorganisms. Figure 6 shows the structures of daphnetin, urolithin A, and coumestrol.

Daphnetin (7,8-dihydroxycoumarin) is isolated from the Chinese herbal plant Daphne Korean Nakai and is known to possess anti-inflammatory, antioxidant, antimalarial, and anti-arthritic activities. It was tested against tyrosine-specific protein kinase EGFR (IC_50_ = 7.67 µM) and serine/threonine kinases PKA (IC_50_ = 9.33 µM) and PKC (IC_50_ = 25.01 µM) [181]. The mechanism of action was demonstrated to be pure ATP-competitive. In the same work, esculin, 2-OH-coumarin, 4-OH-coumarin, and 7-OH-coumarin were examined for their potential as EGFR, PKA, and PKC inhibitors. Surprisingly, these coumarin derivatives did not show any inhibitory effect on EGFR and only slightly on PKC and PKA when tested at a concentration of 200 µM.

Urolithin A is the product of the conversion of ellagic acids and ellagitannins by gut bacteria. It has been described as a potent CK2α inhibitor with an IC_50_ value of 0.31 µM [182]. The urolithin scaffold has been studied as a linkage between the known CK2 inhibitors ellagic acid and 3,8-dibromo-7-hydroxy-4-methylchromen-2-one, resulting in a series of more potent inhibitors through in silico optimization [182].

Coumestrol was found to be a very potent and selective CK2 inhibitor when tested in a panel of several protein kinases. The estimated IC_50_ was found at 228 nM, whereas other kinase activities (DYRK1α, VEGFR, GSK-3β, JAK2) were only reduced by half at a concentration of 10 µM [183]. 

#### 3.1.5. Lignans

**Figure 7:**
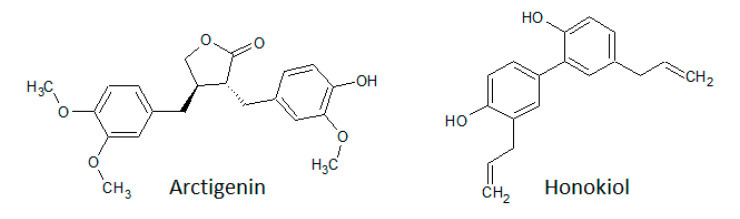
Structures of honokiol and arctigenin.

Lignans are widely produced and found frequently in fiber-rich plants. The structures of two members, honokiol and arctigenin, are shown in Figure 7.

Honokiol is isolated from the leaves and barks of *Magnolia* plants. It displays effects by the inhibition of the PI3K/Akt/mTOR pathway and regulation of the EGFR signaling pathway through suppression of EGFR expression and phosphorylation. Additionally, honokiol inhibits the activation of the mTOR pathway through inhibition of ERK1/2 [184]. Furthermore, it was shown that honokiol downregulates CDK2 and CDK4 in human oral squamous cell carcinoma cells [185].

Arctigenin naturally occurs in plants like *Bardanae fructus*, *Saussurea medusa*, and *Arctium lappa* L. It was shown that arctigenin decreased the phosphorylation of ERK1/2, p38 kinase, and JNK and their enzymatic activities in Raw264.7 cells treated with LPS. The IC_50_ value for MKK1 in vitro was 1 nM [186]. In another study, it was demonstrated that arctigenin interacts with PI3K, and IL-1β-stimulated PI3K and Akt phosphorylation could be inhibited by pre-treatment with arctigenin [187].

#### 3.1.6. Other Polyphenols

**Figure 8:**
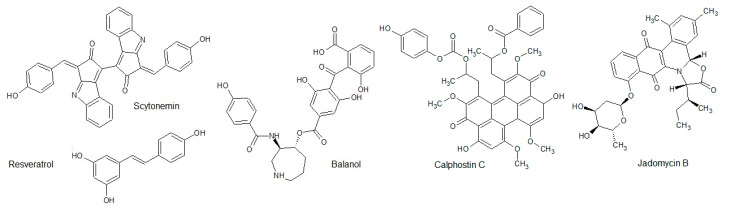
Structures of balanol, scytonemin, resveratrol, calphostin C, and jadomycin B.

As already mentioned, there are many other known polyphenolic substances. Here we describe a few of those not classified in the aforementioned groups. Figure 8 represents the structures of such compounds.

Balanol, isolated from *Verticillium balanoides*, is a fungal secondary metabolite and is described as a very potent ATP-competitive serine/threonine kinases inhibitor. Several classical and novel PKC isoforms were inhibited in the low nanomolar range (IC_50_ = 4–9 nM), and PKCζ belonging to atypical isoforms was inhibited with an IC_50_ of 150 nM [188]. Further analysis showed that balanol was similarly active against other kinases from the AGC, CaMK, and CMGC groups, e.g., protein kinase G (PKG), protein kinase A (PKA), p34cdc2, CaMKII, and MAPK [189]. Balanol also inhibited G protein-coupled receptor (GPCR) kinases (GRK) with IC_50_ values below 1 µM, especially GRK2 (IC_50_ = 42 nM) [190]. The selectivity towards serine/threonine kinases was tested, including two protein tyrosine kinases, the epidermal growth factor receptor (EGFR) kinase, and the Src kinase (pp60 Src). Balanol did not show any effect on these protein kinase activities [191].

Scytonemin is a yellow-green photo-protective pigment isolated from cyanobacteria *Stigonema* sp. [192]. It possesses an inhibitory effect towards several kinases in the low micromolar range, like polo-like kinase 1 (PLK1), PKCβ, Myt1, checkpoint kinase 1, and cyclin-dependent kinase 1/cyclin B. On the other hand, scytonemin is less potent against protein kinase A and Tie2.

Resveratrol belongs to the group of stilbenes and is found in grapes, red wine, olive oil, cranberries, and peanuts. In plants, the role of resveratrol includes the response towards parasitic attack or stress conditions. It has been described as a moderate inhibitor of different tyrosine and serine/threonine protein kinases, including Src, ERK1/2, JNK1/2, p38, PKC, PI3K, PKB, and IKK [193]. One exception is AMPK, which is activated in HepG2 cells in the presence of resveratrol [194]. One of the most promising targets of resveratrol is IKK, which is inhibited in very low micromolar ranges [195]. Another target for resveratrol is the protein kinase C. It has been shown that the compound directly and indirectly affects the phosphorylating activity. Among the tested PKC isoforms, resveratrol only showed effects on the classical isoforms. PKCα was inhibited in a low micromolar range (IC_50_ = 2.0 µM), and the PKCβ1 activity was reduced with an IC_50_ of approximately 105 µM. The novel PKCε and atypical PKCζ could not be affected, even at concentrations of 500 µM. A possible explanation for the diverse effect of resveratrol on PKC isoforms might be the differences in the enzyme structures. Conventional PKCs possess the C1 domain, which is responsible for the binding of resveratrol. Atypical PKCs are lacking this domain, and in novel PKC, the C1 domain differs slightly when compared to conventional PKCs [196].

Calphostin C, isolated from the fungus *Cladosporium cladosporioides*, is a highly selective cell-permeable allosteric PKC inhibitor (IC_50_ = 50 nM) interacting with the regulatory domain [197,198]. It has been shown to exhibit strong inhibitory potential in benign and malignant tumor cell lines through PKC-regulated signal pathways [199]. At much higher concentrations, calphostin C also inhibits myosin light chain kinase, PKA, PKG, pp60v-src protein tyrosine kinase, and DAG kinase. Calphostin C is one of the first described natural allosteric protein kinase inhibitors. 

Jadomycin B is a natural compound produced by bacteria *Streptomyces venezuelae* [200]. Through virtual screening of the Microbial Natural Product Database developed by the Institute of Medicinal Biotechnology, Chinese Academy of Medical Sciences, jadomycin-B was found to have a moderate inhibitory effect on Aurora B activity (IC_50_ = 10.5 µM). In a detailed analysis, it was found that the mode of action was competitive binding to the ATP-site in the catalytic center. In vivo experiments also showed that jadomycin-B blocked the phosphorylation of histone H3 on Ser10 and thereby induced apoptosis in tumor cells not affecting the cell cycle [201].

### 3.2. Indolocarbazole Analogues

Indolocarbozoles are a well-known class of anti-tumor alkaloids. They are capable of inhibiting a wide range of targets within a cell. Their common structural feature is the indolo[2,3-a]pyrrolo[3,4-c] carbazole.

**Figure 9:**
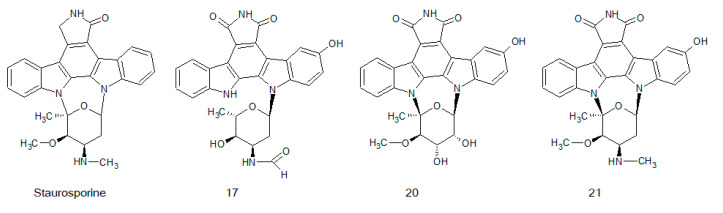
Chemical structures of staurosporine and some of its natural derivatives 17, 20, 21 [202].

Figure 9 shows the chemical structures of staurosporine and naturally occurring derivatives with biological activity.

In 1977, staurosporine was isolated from the bacteria *Streptomyces staurosporeus*. Its biological activities range from antimicrobial up to anti-hypertensive [203]. The first protein kinase identified to be inhibited by staurosporine was PKC with low nanomolar concentrations (IC_50_ = 2.7 nM) [204]. Staurosporine acts as a cell-permeable, potent, reversible, ATP-competitive inhibitor, which possesses a higher affinity towards the ATP-binding site than ATP. In further studies, staurosporine was characterized as an unspecific kinase inhibitor with activities towards a broad range of enzymes. In the nanomolar IC_50_ range (1–20 nM), it also inhibited PKA, PKG, CAMKII, GSK-3β, CDK2, and MLCK. At higher concentrations in the micromolar range, staurosporine was shown to affect the phosphorylation activities of CK1 and CK2, CSK, and MAPK [205,206].

During last years, several new staurosporine derivatives were isolated from *Streptomyces* sp. NB-A13. Other indolocarbazole derivatives were obtained from *Streptomyces* sp. DT-A61, A22, A65, and A68. They possessed inhibitory activity against different PKC isoforms, ROCK2, and BTK in low micromolar or even nanomolar ranges [202].

### 3.3. Furanosteroid Analogues

**Figure 10:**
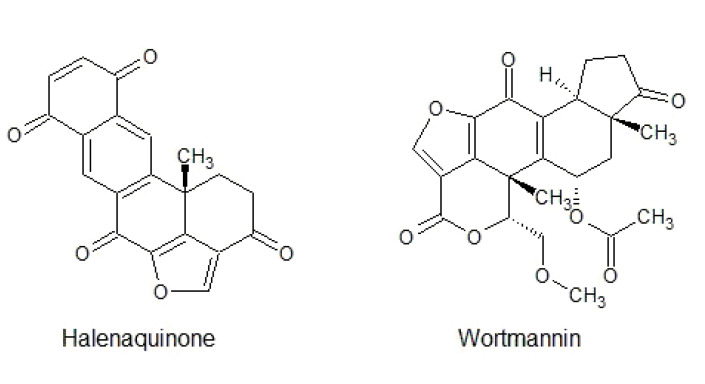
Chemical structures of natural furanosteroid derivatives.

This class of compounds is characterized by a typical steroid structure with an additional furan ring. They are known as kinase inhibitors and for their antimicrobial activities. Figure 10 represents a few members of the furanosteroid class.

Wortmannin is a fungal metabolite first isolated from *Penicillium wortmannii*. It is widely known that it shows inhibitory effects towards PI-3K with an IC_50_ of 2.1 nM. It was previously described as possessing inhibitory potential against MLCK but with an IC_50_ of 170 nM [207]. In further studies, it was determined that other kinases might also be inhibited by wortmannin, e.g., MKK1, PKCi, and Aurora C [208]. Another study showed that wortmannin also very potently inhibits PLK in low nanomolar ranges with an IC_50_ value of 24 nM [209].

Halenaquinone is isolated from the marine sponges *Petrosia alfiani* and *Xestospongia exigua* and *sapra* [210,211]. It was found to be a potent inhibitor of pp60^V-SRC^ with IC_50_ of 1.5 µM [212]. It was also shown that PI-3K was inhibited in a low micromolar range (IC_50_ = 3 µM) [213].

### 3.4. Purine Analogues

**Figure 11:**
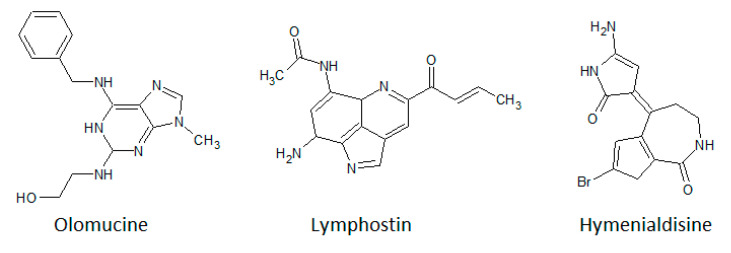
Chemical structures of olomoucine, lymphostin, and hymenialdisine.

Purine analogues have diverse biological activities and are used in the treatment of different cancer types. Figure 11 shows the structure of some members of this class of inhibitors.

Olomoucine is a purine derivative isolated from cotyledons of the radish *Raphanus sativus* L. It was detected as a potent and selective ATP-binding site directed inhibitor of CDK1 (p34^cdc2^)/cyclin B and related kinases [214]. A set of several protein kinases were tested as potential targets of olomoucine [215]. It was shown that p33^CDK2^/cyclin A, p33^CDK2^/cyclin E, and p34^cdc2^/cyclin B were inhibited with an IC_50_ value of 7 µM. Only the activity of p33^CDK5^/p35 was more effectively reduced with an IC_50_ of 3 µM. The effect towards other related CDKs was slightly weaker (p34^cdc2^/cyclinE), or in some cases, it completely failed (p34^CDK4^/cyclin D). Different tested PKC isoforms, PKA, PKG, CaM kinase II, MLC kinase, CK2, EGFR tyrosine kinase, p56^lck^, p56^lyn^, p60^c-src^, and insulin receptor tyrosine kinase, were also not inhibited. 

Lymphostin is a tricyclic aromatic alkaloid that was first isolated from the culture broth of *Streptomyces* sp. KY11783 and identified as an inhibitor of lymphocyte-specific protein tyrosine kinase (LCK) with an IC_50_ value of 50 nM [216,217]. Later, it was revealed that beside LCK, PI-3K was also inhibited with an even lower IC_50_ value of 1 nM [113]. In a further study, lymphostin and its natural derivative neolymphostin showed excellent mTOR inhibition with IC_50_ values of 1.7 nM for lymphostin and 0.8–1.8 nM for the neolymphostins [218].

Hymenialdisine is a constituent of the marine sponges *Hymeniacidon aldis*, *Acanthella aurantiaca*, and *Axinella verrucosa*. Initially, it was characterized as an inhibitor of CDK1, 2, and 5, GSK-3β, and CK1 with IC_50_ values of 22, 40–70, 28, and 35 nM, respectively [32]. Furthermore, hymenialdisine was also identified as a potent MEK1 inhibitor with an IC_50_ of 6 nM, whereas several other kinases were inhibited only in the micromolar range, e.g., Aurora-A, Her1/2, IKKα, PKA, and PKB [33].

### 3.5. Other Natural Substances

**Figure 12:**
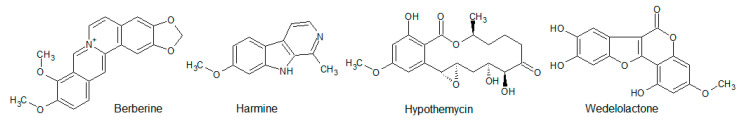
Chemical structures of other natural protein kinase inhibitors.

Beside those natural inhibitor classes described above, there exist much more other substances not classified in these. Few of them are mentioned in this chapter and their structures are shown in Figure 12.

Hypothemycin was first isolated from *Hypomyces trichothecoides*. Belonging to the family of resorcylic acid lactones, it showed only weak anti-fungal and anti-malaria activity [219,220]. In a study with 124 protein kinases, it decreased the enzyme activities in an ATP-competitive manner. Several kinases were inhibited in a low micromolar range, e.g., ERK1/2, PDGFRα, PDGFRβ, SRC, PKD1, TRKA, and TRKB. Hypothemycin showed high potential effects towards MEK1/2, FLT-3, and VEGFR1/2 with Ki values in low nanomolar ranges [221]. 

The tricyclic β-carboline alkaloid harmine is found in the harmal plant and acts as a monoamine oxidase A inhibitor with anti-depressive, analgesic, and anti-bacterial effects. It was shown that harmine was a potent and selective inhibitor for DYRK1A with an IC_50_ value of 33 nM, whereas the inhibition constants for DYRK1B, DYRK2, and DYRK4 were 166 nM, and 1.93 and 79.75 µM, respectively [222]. In further selectivity studies, the potential of harmine was also examined against other protein kinases. The results for PIM3 and CK1 were in a similar range to that of DYRK2, with IC_50_ values of 4.3 and 1.5 µM, respectively [208]. In another study, the panel of tested kinases was extended up to 402 different enzymes [223]. Within this huge amount, only a few kinases were found to be inhibited by harmine in a micromolar range, e.g., CLK and HIPK. 

Wedelolactone is classified as a coumestan and occurs in *Eclipta alba* and *Wedelia calendulacea*. It is described as a cell-permeable, selective, and reversible IKKα/β inhibitor with an IC_50_ value in the low micromolar range. Wedelolactone is a specific inhibitor, and until now, no inhibitory effects against other protein kinases have been described [224].

## 4. Conclusions

Natural sources, like plants or microorganisms, provide a huge pool to identify and isolate new compounds which possess biological activities. They have been known for thousands of years and used in traditional medicine. Naturally occurring substances are able to modulate numerous signaling pathways, cell proliferation, and angiogenesis. Besides this, they possess antimicrobial, antioxidant, and anti-inflammatory activities. Protein kinase inhibitors are receiving broader interest in the search for new drugs. A variety of kinases are targeted by natural compounds, including all subfamilies from the kinome. There are numerous compounds that are promising candidates for effective drugs against severe diseases. 

The negative aspects of most natural products are their high molecular weight, poor stability, and often weak solubility. Computer-aided research will be useful in the development of more potent and selective protein kinase inhibitors to overcome these negative physico-chemical characteristics of the compounds through the synthesis of better derivatives.

Protein kinase inhibitors are often unspecific and, therefore, active against a high number of different kinases. This is often due to their mechanism of action as reversible ATP-competitors, especially in the case of polyphenols. There are few substances isolated from bacteria or fungi which possess an irreversible binding mode, like calphostin C, wortmannin, halenaquinol, and hypothemycin. Therefore, it is necessary to modify those substances to get more specific compounds. As shown in experiments on cell lines, those kinase inhibitors often interact with other targets in the cell rather than with kinases. These might be DNA topoisomerases, MMPs, caspases, and phosphatases. 

## Figures and Tables

**Table 1:** Selected ePKs and their cellular functions.

ePK	Function in the Cell	Ref.
Akt/PKB	- Glucose storage regulation in the form of glycogen by phosphorylation of GSK3. - Regulation of cell survival via phosphorylation of ASK1. - Regulation of many processes, including metabolism, proliferation, cell survival, growth, and angiogenesis.	[10,11]
AMPK	- Control of food intake. - Key role in carbohydrate and lipid metabolism in skeletal muscle. - Promotion of catabolic pathways to generate ATP production and inhibition of anabolic pathways. - Negative regulation of mTOR signaling.	[12,13]
ASK1	- Positive regulation of vascular smooth muscle proliferation. - Crucial role in the apoptosis signal transduction pathway through mitochondria-dependent caspase activation required for sustained activations of JNK/p38 MAP kinases and apoptosis.	[14]
CDKs	- Regulation of cell cycle and cell division. - Modulation of transcription in response to several extra- and intracellular cues.	[15,16,17]
CK2, CK2α	- Positive regulation of cell growth and proliferation. - Stimulation of Wnt signaling pathway. - Regulation of signal transduction by p53 mediator. - Repression of cysteine-type endopeptidases involved in apoptosis. - Negative regulation of ubiquitin-dependent protein degradation.	[18,19,20]
EGFR	- Regulation of angiogenesis, cell motility, differentiation, proliferation, and survival. - Inhibition of apoptosis. - Positive regulation of NFκB signaling. - Activation of major downstream signaling cascades, including the RAS-RAF-MEK-ERK, PI3K-Akt, PLCγ-PKC, and STATs modules.	[21,22,23,24]
ERK1/2	- Positive regulation of gene expression and translation. - Regulation of proliferation, differentiation, and survival. - Regulation of apoptosis and stress response.	[25,26]
FAK1	- Positive regulation of PI3K and Akt/PKB signaling. - Positive regulation of cell population proliferation and cell migration. - Regulation of cell adhesion mediated by integrin.	[27,28]
FGFR	- Cell proliferation, differentiation, maturation, and migration. - Selective apoptosis during embriogenesis. - Stimulation of MAPK, PI3K, and Akt/PKB signaling pathways.	[29,30,31]
GSK3β	- A negative regulator in the hormonal control of glucose homeostasis, Wnt signaling, and the regulation of transcription factors and microtubules by inactivation of glycogen synthase, eIF2B, β-catenin, APC, JUN, and others. - Negatively regulates extrinsic apoptotic signaling pathway via death domain receptors.	[32]
IGF1-R	- Regulation of cell growth and survival, suppression of apoptosis. - Inactivation of MAPKK activity. - Stimulation of MAPK, PI3K, and Akt/PKB signaling pathways. - Positive regulation of cell population and migration. - Regulation of JNK cascade.	[33,34]
IKKα	- Essential for activation of members of the NF-κB family of transcription factors, which play a fundamental role in lymphocyte immunoregulation. - Positive regulation of transcription by RNA pol II.	[35,36,37,38]
JAK1-3	- Regulation of receptor signaling pathway via JAK-Stat. - Mediates interleukin-7-induced activation of PI3K.	[39,40]
JNK	- JNK phosphorylates heats shock transcription factor-1 (HSF-1) and suppresses its transcriptional activity. - Stimulation of protein insertion into mitochondrial membrane involved in apoptotic signaling pathway.	[41,42,43]
Lck	- Activation of cysteine-type endopeptidase activity involved in apoptosis. - Stimulation of Akt/PKB signaling. - Lymphocyte activation and stimulation of leukocyte cell adhesion. - Regulation of cell proliferation.	[44,45,46]
mTOR	- mTORC1 complex functions as a nutrient/energy/redox sensor and controls protein synthesis. - mTORC2 complex regulates actin cytoskeleton and Akt/PKB activity, thus affecting metabolism and survival. - Promotion of activation of IGF-IR and InsR.	[47,48,49,50]
PDK1	- Phosphorylation of activation loops and activation of protein kinases: Akt/PKB, RSKs (p70 and p90S6K), SGK, PKC, p21-activated kinase (PAK), and polo-like kinase 1.	[51]
	- Regulation of PI3k/Akt/mTOR, Ras/MAPK, and c-Myc signaling pathways.	
PKA	- Regulation of metabolic pathways, cell cycle, proliferation, and differentiation. - Regulation of translation on the elongation step.	[52,53]
PKC	- Signal transduction in mediating the effects of many extracellular stimuli, including growth factors, hormones, and drugs.	[54,55]
PKG	- Regulation of apoptosis and survival in neural cells. - Regulation of platelet activation and adhesion, smooth muscle contraction, blood pressure, cardiac function, gene expression, feedback of the NO-signaling pathway, and hippocampal and cerebellar learning.	[56,57]
Src	- Control of many functions, including cell adhesion, growth, movement, and differentiation. - Regulation of embryonic development and cell growth. - Suppression of cysteine-type endopeptidase activity involved in the apoptotic process. - Activation of Akt/PKB activity.	[58,59]
VEGFR1/3	- Plays an essential role in the regulation of angiogenesis, vascular development, vascular permeability, and embryonic hematopoiesis. - Promotes proliferation, survival, migration, and differentiation of endothelial cells. - Promotes reorganization of the actin cytoskeleton.	[60,61,62]

**Table 2:** Examples of natural products affecting protein kinase activity.

Compound	Target	Biological Effect	Ref.
**Tyr Protein Kinases**			
**Alpinetin**	**Stat3↓**	Altered protein expression levels of cyclin-D1, c-Myc, survivin, Bcl-2, Bax, TIMP-1, TIMP-2, MMP-2, MMP-9, as well as cleaved caspase-3 and PARP in SKOV3 cells.	[101]
**Auriculasin**	**VEGFR2↓**	Inhibition of angiogenesis by modulating VEGFR2-related signaling pathways. Inhibition of VEGFR2 activation, as well as phosphorylation of intracellular downstream protein kinases AKT, mTOR, PI3K, p38, ERK, and Src.	[102]
**Cantharidin**	**JAK1/** **Stat3↓**	Suppression of VEGF-induced activation of Stat3 and inhibition of JAK1 and ERK phosphorylation.	[103]
**Curcumin**	**IGF-1R↓**	Inhibition of phosphorylation: IGF1R, IRS1, AKT, S6K, and 4EBP1 in the mouse keratinocyte cell line.	[104,105]
	**Src↓,** **PTK2↓**	Inhibition activity of v-Src led to a reduction of Src-Tyr phosphorylation, decreased Src-mediated Shc phosphorylation, ERK activation, and cell proliferation in v-Src transformed cells.	[106]
**Emodin**	**JAK2↓**	Inhibition of IL-6-induced JAK2/Stat3 pathway induced apoptosis.	[107]
	**Her2/neu↓**	Suppression of Her2/neu PTK activity and proliferation; repression of transformation and metastasis.	[108]
**Honokiol**	**EGFR↓**	Inhibition of U251 and U-87 MG human glioma/glioblastoma cell viability, colony formation, and promoted apoptosis. Inhibition of cell migration/proliferation and invasion. Induction of apoptosis and reduction of Bcl-2 expression, accompanied by an increase in Bax expression. Reduced expression of EGFR, CD133, and nestin. Suppression of AKT and ERK signaling pathway activation.	[109]
**Luteolin**	**VEGF/** **VEGFR2↓**	Decreased VEGF, cell migration, and viability of triple-negative breast cancer cell lines MDA-MB-435.	[110,111]
	**EGFR↓**	Inhibition of EGF-induced activities of EGFR signaling pathway in human breast cancer cell lines and PI3K/AKT, MAPK/ERK1/2, Stat3 signal pathways.	[112]
**Lymphostin**	**Lck↓**	Inhibition of Src family kinase Lck activity in Jurkat T cells.	[113]
**Quercetin**	**JAK2/Stat3↓**	Inhibition of hepatocellular carcinoma progression by modulating cell apoptosis, migration, invasion, and autophagy. Effects partly related to the JAK2/Stat3 signaling pathway.	[114]
**Tannic acid**	**EGFR↓ Stat1/3↓**	Tannic acid binding to EGFR inhibited the tyrosine kinase activity, modulated the EGFR/JAK2/Stat1/3 and p38/ Stat1/ p21WAF1/CIP1 pathways, and induced G1-arrest and intrinsic apoptosis in breast carcinomas.	[115]
**Ser/Thr Protein Kinases**			
**β-elemene**	Cdc2↓	Cell cycle G2/M phase was arrested in A2780 and A2780/CP human ovarian carcinoma cells in vitro, mediated by alterations in cyclin and CDK expression, the down-regulation of Cdc2, cyclin A, and cyclin B1, and the upregulation of p21WAF1/CIP1 and p53 proteins.	[116]
**Acacetin** **Genkwanin** **Isorhamnetin**	Akt/PKB↓	Cell cycle arrested at G2/M as a result of PI3Kγ inhibition and inactivation of PI3K, AKT, mTOR, p70RSK, and ULK, resulting in apoptosis in human breast cancer cells.	[117]
**Alpinetin**	ERK↓	Phosphorylation of IκBα protein, p65, p38, and ERK inhibited in LPS stimulated RAW 264.7 cells.	[118]
**Apigenin**	CK2α↓	Inactivation of CK2α resulted in inhibition of the sphere-forming cell capacity of HeLa.	[119]
	IKKα↓	Direct binding with IKKα attenuated kinase activity and suppressed NF-ĸB/p65 activation in human prostate cancer PC-3 and 22Rv1 cells.	[120]
**Artemisinin**	IKKα↓	Exhibited anti-inflammatory activities in TPA-induced skin inflammation in mice; inhibited the expression of TRAF2 and RIP1; inhibited TNFα induced NF-κB reporter gene expression, phosphorylation, and degradation of IκBα, and p65 nuclear translocation.	[121,122]
**Baicalin**	PKC↓, MAPK↓	ROS production reduced, suppressed Casp3 cleavage for inducing apoptosis. Inhibited activation of PKC/MAPK signaling pathway for down regulating JNK, p38, ERK, PKCα, and PKCδ in piglet monocytes stimulated by *Haemophilus parasuis*.	[123]
**Berberine**	AMPK↑, mTOR↓	AMPK activated, as a major regulator of metabolic pathways, mTOR inhibited. mTOR targets: 4EBP1 and p70RSK down-regulated.	[124]
	MLCK↓	Reduced amplitude of contraction in isolated duodenum and gastric strips in rats by inhibition of MLCK and down-regulation of MLC20 and Mg^2+^-ATPase activity.	[125]
**EGCG**	RAF↓, MEK1/2↓, ERK1/2↓	Reduced protein levels of pEGFR, H-RAS, p-RAF, p-MEK1/2, and pERK1/2 in human thyroid carcinoma cells. Inhibition of the growth by induced apoptosis and down-regulated angiogenesis.	[126]
**Emodin**	CK2↓	CK2 inhibition cancer cells to Fas and TRAIL ligand-induced apoptosis. CK2 inhibition enhanced the cytotoxicity of natural killer cells HepG2 and Hep3B in vivo.	[127]
**Genistein**	PLK1↓	In human neuroblastoma SK-N-MC cells, the protein level of MDC1, p53, p21WAF/CIP1, and Bax increased in a dose-dependent manner. Phosphorylation of Chk2 and Cdc25C increased. In addition, consistent with PLK1 down-regulation, Cdc25C phosphorylation inhibited at Ser-198. Down-regulation of proteins Chk2, Cdc25C, Cyclin B1, and Cdc2 as well as Bcl-2 resulted in neuronal apoptosis and G2/M cell cycle arrest.	[128]
**Hibiscone C**	PI3K↓	Hibiscone C competitively inhibited PI3K activity in intact cells, slowed proliferation, and induced cell death.	[129]
**Luteolin**	p90RSK↓, JNK1↓,	Luteolin exhibited anti-photoaging effects in vitro and in vivo by suppression of JNK1 and p90RSK activity and may have potential as a treatment for the prevention of skin aging.	[130]
**Quercetagetin**	PIM1↓	PIM1 activity in intact RWPE2 prostate cancer cells inhibited in a dose-dependent manner. RWPE2 cells showed pronounced growth inhibition at inhibitor concentrations that blocked PIM1 kinase activity. The ability of quercetagetin to inhibit the growth of other prostate epithelial cell lines varied in proportion to their levels of PIM1 protein.	[131]
**Quercetin**	Akt/PKB↓, ERK↓	Akt/PKB and ERK inhibited, resulting in reduced phosphorylation of BAD and a strong activation of caspase-3.	[132]
	CK2↓, Akt/PKB↓	CK2 and PI3K/Akt pathways inhibited in chronic lymphocytic leukaemia HG3 cells.	[133]
**Resveratrol**	PKC↓, MAPK↓, IKKβ↓	TPA-induced expression of PKC inhibited in human mammary and oral epithelial cells PKCδ in human cervical cancer and affected PKC activity, inducing apoptosis in human colon carcinoma cells. Activity of kinases: PKC, MAPK, IKKβ, and transcription factors: Stat3, HIFα, NF-κB, AP-1, were repressed, resulting in various responses to oncogenic stimuli.	[134,135,136,137]
**Scutellarein**	PKC↓	Platelet adhesion and aggregation induced by multiple G protein coupled receptor agonists, such as thrombin, was inhibited in a concentration-dependent manner. Scutellarein had a mild effect on intracellular Ca2+ mobilization and cAMP levels. The role of scutellarein as PKC inhibitor was confirmed by PKC activity analysis and molecular docking with PKCα and β.	[138]
	PKC βI↓, PKC βII↓, PKCδ↓	PKC activity in the membrane fraction of thoracic aorta smooth muscle cells of diabetic rats inhibited. The translocation inhibition of PKC in vivo and in vitro in diabetic rats may have value as a drug in the treatment of diabetic complications via its inhibition of PKC βI, βII, and δ.	[139]
**Songorine**	GSK3β↓	Cell growth and metastasis in epithelial ovarian cancer suppressed via the GSK3β/β-catenin and Bcl2/Bax signaling pathways.	[140]
